# Facile Fabrication of Magnetic Metal-Organic Framework Composites for the Highly Selective Removal of Cationic Dyes

**DOI:** 10.3390/ma11050744

**Published:** 2018-05-07

**Authors:** Huijun Li, Qingqing Li, Yaling He, Ning Zhang, Zhouqing Xu, Yuan Wang

**Affiliations:** College of Chemistry and Chemical Engineering, Henan Polytechnic University, Jiaozuo 454000, China; lihuijunxgy@hpu.edu.cn (H.L.); lq18339169060@163.com (Q.L.); heyaling1220@163.com (Y.H.); zhning996@163.com (N.Z.)

**Keywords:** magnetic composite material, core-shell structure, the capsulation of cationic dyes, electrostatic attractions

## Abstract

In this work, we show a novel magnetic composite material **Fe_3_O_4_@HPU-9** (**HPU-9** = {[Cd(L)_0.5_(H_2_O)](DMA)(CH_3_CN)}_n_) (H_4_L = 1,1′-di(3,5-dicarbonylbenzyl)-2,2′bimidazoline, DMA = *N*,*N*-dimethylacetamide) constructed by in situ growth of **HPU-9** on Fe_3_O_4_, which has excellent absorption of cationic dyes from aqueous solution. The **Fe_3_O_4_@HPU-9** particle possesses a well-defined core-shell structure consisting of a Fe_3_O_4_ core (diameter: 190 nm) and a **HPU-9** shell (thickness: 10 nm). In the composite, the **HPU-9** shell contributes to the capsulation of cationic dyes through electrostatic attractions between **HPU-9** and cationic dyes, while the Fe_3_O_4_ core serves as magnetic particle. The maximum absorption capacity of **Fe_3_O_4_@HPU-9** for R6G was 362.318 mg·g^−1^. The absorption kinetics data were well described by a psedo-second-order model (R^2^ > 0.99), and the equilibrium data were also well fitted to Langmuir isotherm model (R^2^ > 0.99). Our data confirmed that the proposed magnetic composite could be recycled and reused several times without centrifugal separation, making it more convenient, economic and efficient than common adsorbents.

## 1. Introduction

Dyes are currently widely used in various industries, such as printing, wool, paper, nylon and silk [1,2]. However, the emission of dyes into the environment has raised widespread public concern relating to water pollution and human health [3,4,5,6]. Numerous conventional porous materials, such as zeolites, polymeric resins, carbon materials and mesoporous silica, have been considered for dye absorption but exhibit weak selectivity toward targeted dyes and are not cost-effective for practical application [7,8,9]. Previous reports have demonstrated that metal-organic frameworks (MOFs) show excellent performances in the recognition capability and selectivity toward a variety of organic dye pollutants because of their tunable chemical functionality, pore microenvironment, structural diversity and high surface areas [10,11]. Principally, the size dimension and property of the pores in the MOFs play important roles in the selective absorption of targeted dyes [12,13]. First, MOFs possessing a relatively large pore size are the necessary prerequisite to selective dye absorption. Second, the interactions between dye molecules and absorption sites on the pore walls of MOFs, which could improve the physical and/or chemical absorption capability of targeted dye molecules, are the guarantee of the success of removing targeted dyes. In this article, we demonstrated that the utilization of a large vacant pore space with anionic binding sites can improve cationic dye uptake through direct electrostatic attractions between adsorbent and adsorbate moieties. 

Although MOFs have purely microporous structures that offer high-affinity binding sites for targeted dyes, they are limited by significant defects, such as powder morphology, difficulty of separation and poor stability, which reduce the efficiency and recyclability of adsorbents in practice. To address this problem and maximize the dye absorption capability of MOFs, nanoparticles with unique magnetic properties could be incorporated into MOFs [14,15]. Magnetic separation based on Fe_3_O_4_ is a considerably convenient, economic and efficient approach [16,17]. Thus, we proposed a cationic dye absorption method with magnetic anionic MOF composites that can be magnetically separable and are conveniently reusable. To the best of our knowledge, few studies have reported on the use of magnetic MOF composites for dye removal from aqueous solution. Herein, we successfully synthesized a new porous anionic MOF, namely, {[Cd(L)_0.5_(H_2_O)](DMA)(CH_3_CN)}_n_ (**HPU-9**), with a large 1D channel along the *c* axis. The anionic channel enables the encapsulation of cationic dyes through host-guest interactions. In addition, a magnetic **Fe_3_O_4_@HPU-9** hybrid was prepared through a hydrothermal method by in situ growth of **HPU-9** on Fe_3_O_4_. The crystalline structure and composition of Fe_3_O_4_ and **Fe_3_O_4_@HPU-9** were identified by TEM, SEM, PXRD, IR and ICP characterization. The maximum absorption capacity of **Fe_3_O_4_@HPU-9** for R6G was 362.318 mg·g^−1^. The absorption kinetics data were well described by a psedo-second-order model (R^2^ > 0.99) and the equilibrium data were also well fitted to Langmuir isotherm model (R^2^ > 0.99). As a result, the absorption and reusability of the magnetic **Fe_3_O_4_@HPU-9** hybrid for dyes suggested that the magnetic hybrid could be employed as a convenient, economic and efficient adsorbent for the treatment of wastewater containing cationic dyes.

## 2. Materials and Methods

All chemicals were commercially available and used as purchased. The detailed information is listed in Table S1 in the Supplementary Materials. Infrared (IR) data were recorded on a BRUKER TENSOR 27 spectrophotometer (BRUKER OPTICS, Munich, Bavaria, Germany) with KBr pellets in the region of 400–4000 cm^−1^. Elemental analyses (C, H and N) were carried out on a Flash EA 1112 elemental analyzer (Suzhou Orco Metrology instrument Co., Ltd., Suzhou, China). Powder X-ray diffraction (PXRD) patterns were recorded using CuKα radiation on a PANalytical X’Pert PRO diffractometer (PANalytical B.V., Almelo, The Netherlands). Thermogravimetric analysis (TGA) was recorded on a Netzsch STA 449C thermal analyzer (NETZSCH, Selb, Bavaria, Germany) between 30 and 800 °C at a heating rate of 10 °C·min^−1^ in atmosphere. The UV spectra were recorded on a Purkinje General TU-1800 spectropho-tometer (Beijing Purkinje General Instrument, Beijing, China). The morphologies and microstructures of the as-synthesized samples were characterized by field-emission scanning electron microscopy (SEM) (S-4800, Hitachi, Chiyoda, Japan) and transmission electron microscopy (TEM) (JEM-1200EX, JEOL Ltd., Tokyo, Japan). Surface elements of samples were mapped with electron disperse spectroscopy (EDS) equipped with SEM. The amount of Fe^3+^ and Cd^2+^ ions was determined by using an HK-2000 (Beijing Huake-Yitong Analytical Instruments Co., Ltd., Beijing, China) inductively coupled plasma.

Synthesis of {[Cd(L)_0.5_(H_2_O)](DMA)(CH_3_CN)}_n_. **HPU-9** was synthesized via a hydrothermal method. Cd(NO_3_)_2_ (9.2 mg) and 4.9 mg of H_4_L were dissolved in 5 mL of anhydrous DMA, CH_3_OH and CH_3_CN (2:2:2) in a 25 mL Teflon liner and kept at 80 °C for 72 h. Colorless **HPU-9** was obtained; yield: 47%. Elemental analysis data calculated (calcd.) for C_18_H_21_CdN_4_O_6_: C 43.08% H 4.21 % N 11.16%. Found: C 43.77, H 4.16, N 11.32%.

Preparation of Fe_3_O_4_ particles. The Fe_3_O_4_ particles were synthesized via a conventional solvothermal reaction. Specifically, 1 g FeCl_3_·6H_2_O was dissolved in 30 mL ethylene glycol, mixed with 2.7 g NaAc and 0.75 g polyethylene glycol at room temperature and stirred for 30 min to obtain a uniform mixture and then reacted in a Teflon liner at 200 °C for 8 h. The obtained solid was washed several times with C_2_H_5_OH and H_2_O and finally dried at 60 °C *in vacuo* for 24 h.

Preparation of magnetic porous **Fe_3_O_4_@HPU-9** core-shell particles: 9.2 mg Cd(NO_3_)_2_ was dissolved in 4 mL CH_3_OH and CH_3_CN (2:2) and then mixed with 4.9 mg of H_4_L dissolved in 2 mL of anhydrous DMA. The mixture was stirred for 30 min, and 2 mg Fe_3_O_4_ was added and then transferred to a 25 mL Teflon liner and kept at 80 °C for 72 h. Subsequently, the reaction mixture was centrifuged, and the composite materials were collected and washed several times with water.

Crystal Data Collection and Refinement. The crystallographic diffraction data for **HPU-9** were obtained on a Siemens Smart CCD single-crystal X-ray diffractometer (Germany bruker Ltd., Karlsruhe, Germany) with a graphite monochromatic MoKα radiation (λ = 0.71073 Å) at 293 K. The structure was solved by direct methods using the SHELXS-2014 program of the SHELXTL package and refined on *F*^2^ by full-matrix least-squares techniques with SHELXL-2014. All empirical absorption corrections were applied using the SADABS program. All non-hydrogen atoms in the crystal structure were refined with anisotropic thermal parameters. The crystallographic data and structural refinement parameters of the complex are summarized in Table S2. CCDC 1812922 for **HPU-9**.

## 3. Results and Discussion

### 3.1. Structural Description

**Figure 1:**
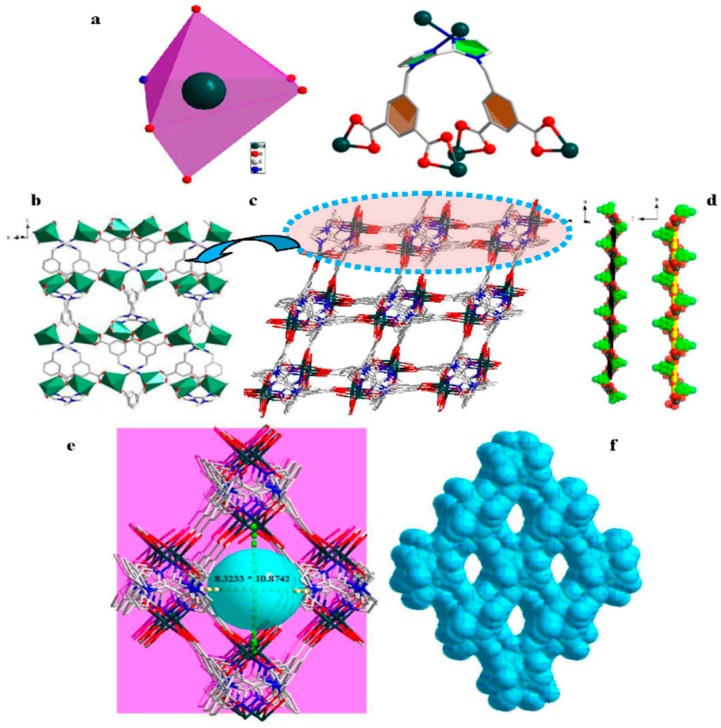
(**a**) The coordination environments of the ligand and Cd atom; (**b**) the two-dimensional layer; (**c**) the three-dimensional network; (**d**) the left- and right-handed helical chains; (**e**) the 1D channel; (**f**) the space-filling of **HPU-9**.

Single crystal X-ray diffraction analysis reveals that **HPU-9** crystallizes in the orthorhombic space group Pccn and displays a 3D porous network structure. The asymmetric unit of **HPU-9** consists of one crystallographically independent Cd^2+^ ion, half H_4_L ligand, as well as one H_2_O coordinated molecule, one CH_3_CN molecule and one DMA guest molecule. The Cd center is six-coordinated by five oxygen atoms and one nitrogen atom in a distorted octahedral [CdO_5_N] geometry. The Cd-O distances are in the range of 2.258–2.377 Å. Each L^4−^ ligand serves as a µ^6−^ bridge connecting with six Cd^2+^ atoms, in which the four carboxylate groups exhibit the same µ_1_-η^1^:η^1^ chelate coordination mode (Figure 1a). Through the inter-connnection of Cd^2+^ and L^4−^, a two dimensional layer is formed, as shown in Figure 1b. It is interesting to note that there are two different helical chains, left- and right- helical chains (Figure 1d), and due to the existence of the benzene and trizole ring, the two dimensional layers are connected with each other, forming a three-dimensional open framework imparting nanoscale quadrangle, as shown in Figure 1c. As evidenced from the single-crystal X-ray diffraction analyses, the quadrangle window in **HPU-9** shows a pore size of ca. 8.3 × 10.9 Å^2^ (Figure 1e,f). The PLATON program indicates that the vacant space in **HPU-9** is approximately 40.2% (1446.5/3601.0 Å^3^). The TG curve of **HPU-9** is shown in Figure S1. A weight loss of 28.06% (calcd, 28.96%) is detected in the temperature range of 50–180 °C, corresponding to the loss of guest molecules and coordinated water molecules. Finally, the white CdO residue constitutes 26.11% (calcd, 25.36%). The BET surface area of **HPU-9** and **Fe_3_O_4_@HPU-9** is 312 and 299 m^2^·g^−1^.

### 3.2. The Hybrization of **HPU-9** and Fe_3_O_4_

**Scheme 1:**
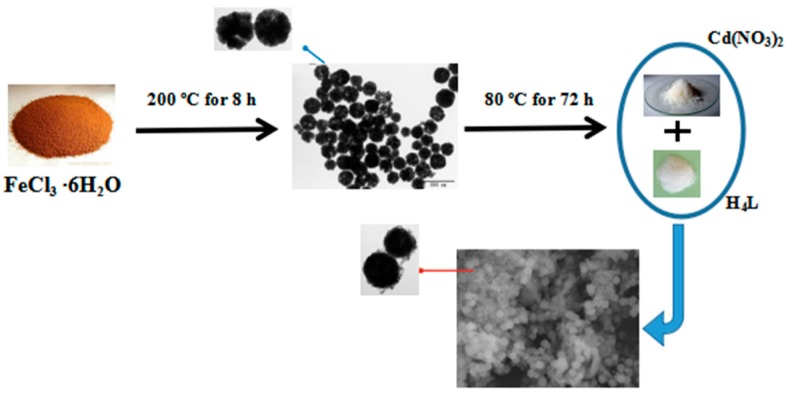
Synthesis of Fe_3_O_4_@HPU-9.

Dyes are very widely used in various industries such as cosmetics, printing and paper [18,19]. Dye removal from wastewater has led to tremendous environmental pollution, which has raised public concern [7,8,9]. Nonetheless, defects such as the difficulty of separation exist in the dye removal process when **HPU-9** is used as an absorbent. Magnetic hybrid materials used in wastewater treatment are very practical [20,21,22]. Magnetic separation based on the superparamagnetic Fe_3_O_4_ was obviously more efficient, economic and convenient [23,24]. Thus, a magnetic **Fe_3_O_4_@HPU-9** hybrid was synthesized through the hydrothermal method (Scheme 1). The crystalline structure and composition of Fe_3_O_4_ and **Fe_3_O_4_@HPU-9** were identified by TEM, SEM, PXRD, IR and ICP characterization.

**Figure 2:**
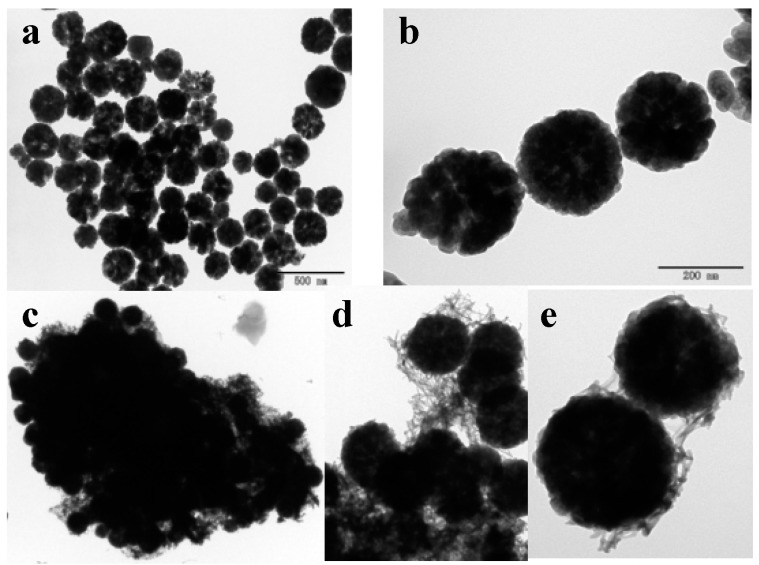
TEM images of Fe_3_O_4_ (**a**,**b**) and **Fe_3_O_4_@HPU-9** (**c**–**e**).

**Figure 3:**
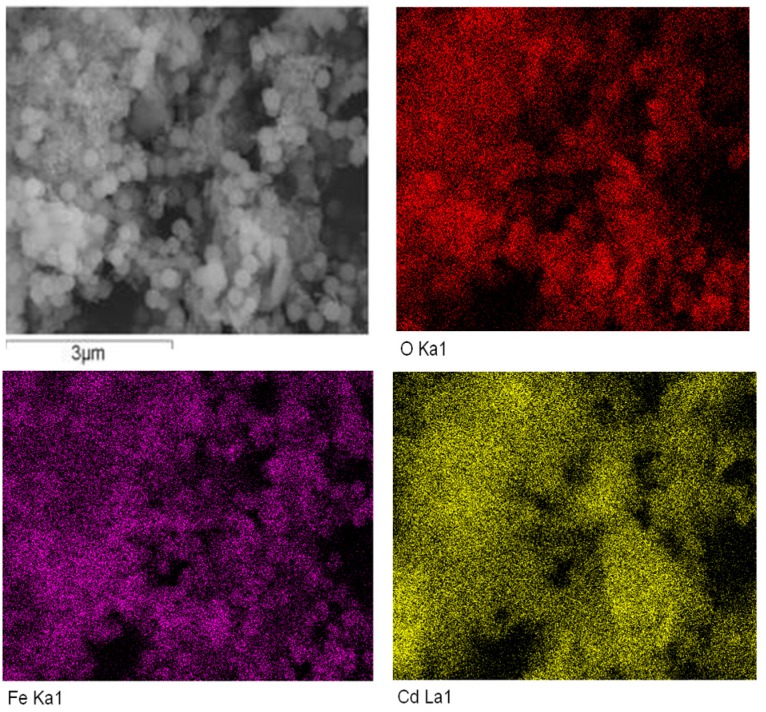
The SEM images of **Fe_3_O_4_@HPU-9** and the corresponding elemental mapping of the composite.

The TEM image of **Fe_3_O_4_@HPU-9** showed that the Fe_3_O_4_ particles were wrapped with a **HPU-9** layer consisting of numerous small crystals (Figure 2). The **Fe_3_O_4_@HPU-9** particles possessed a well-defined core-shell structure consisting of a Fe_3_O_4_ core (diameter: 190 nm) and a **HPU-9** shell (thickness: 10 nm). The SEM results revealed that the **Fe_3_O_4_@HPU-9** had spherical morphology (Figure 3). The elemental mapping of **Fe_3_O_4_@HPU-9** revealed the distribution of Fe, Cd, O within the structures. In particular, Fe and Cd were almost uniformly distributed in **Fe_3_O_4_@HPU-9**. The ICP result showed that the ratio of Fe:Cd was 1.83:1. The TGA curve of **Fe_3_O_4_@HPU-9** is also given in Figure S1. The initial weight loss in the temperature range of 50–180 °C is attributed to the release of CH_3_CN, H_2_O and DMA (observed, 21.83, calcd, 22.69%). From 400 °C, the framework begins to collapse. The Fe_2_O_3_ and CdO residues of 42.33% (calcd, 41.03%) are observed.

**Figure 4:**
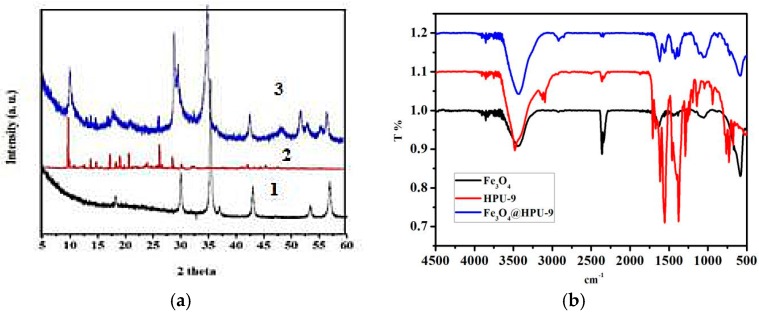
(**a**) The PXRD patterns of Fe_3_O_4_ (1); **HPU-9** (2) and **Fe_3_O_4_@HPU-9** (3); (**b**): the FT-IR spectra of Fe_3_O_4_, **HPU-9** and **Fe_3_O_4_@HPU-9**.

The PXRD patterns of Fe_3_O_4_, **HPU-9** and **Fe_3_O_4_@HPU-9** are shown in Figure 4a. The diffraction peaks of Fe_3_O_4_ and **HPU-9** were observed in **Fe_3_O_4_@HPU-9**, thereby indicating the successful hybridization of **Fe_3_O_4_@HPU-9**. The IR spectra of Fe_3_O_4_, **HPU-9** and **Fe_3_O_4_@HPU-9** are also provided in Figure 4b. The carboxylate groups in H_4_L were observed at 1383 and 1409 cm^−1^. All of the characteristics peaks of **HPU-9** were also observed in **Fe_3_O_4_@HPU-9**, confirming that the structure of **HPU-9** was preserved. Therefore, considering that the results were supported by the experiments, we concluded that the **Fe_3_O_4_@HPU-9** composite that consists of Fe_3_O_4_ and **HPU-9** was successfully synthesized.

### 3.3. Dye Absorption and Separation

**Figure 5:**
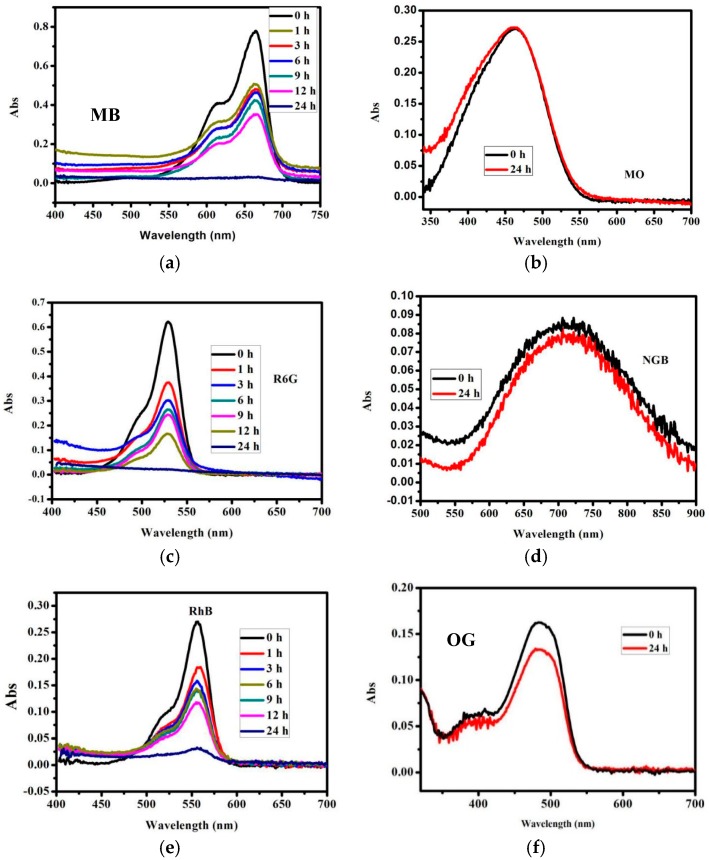
UV-vis spectra of aqueous solutions of MB (**a**); R6G (**b**); RhB (**c**); MO (**d**); NGB (**e**); OG (**f**) before and after 24 h in the presence of **Fe_3_O_4_@HPU-9**. Inset: Photographs of tracing the dye-absorption process through immersing the as-prepared crystals of **Fe_3_O_4_@HPU-9** in aqueous solutions of different dyes.

**Figure 6:**
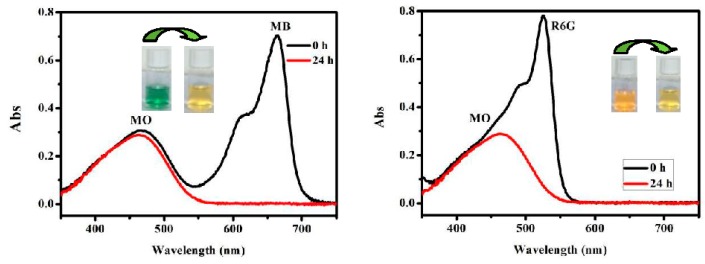

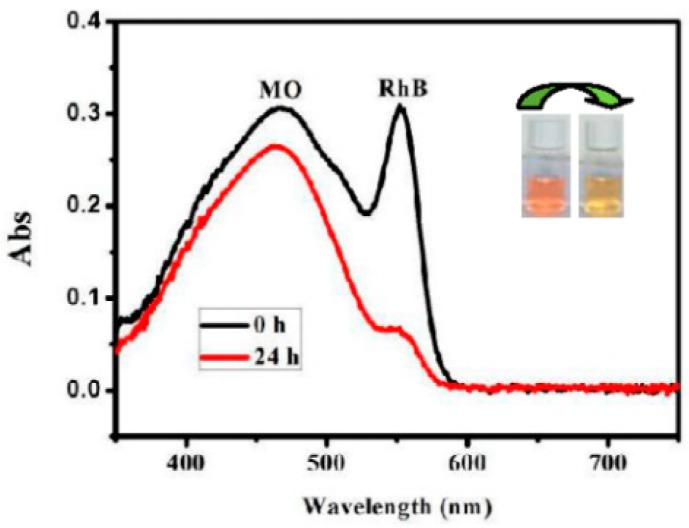
The selective absorption of cationic dye from the mixtures of cationic and anionic dye solutions by **Fe_3_O_4_@HPU-9**.

**Figure 7:**
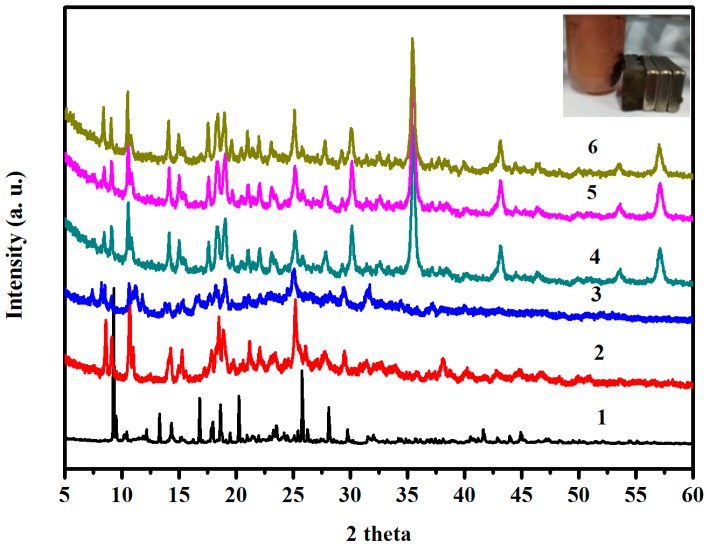
Powder X-ray diffraction (PXRD) patterns of **HPU-9 and Fe_3_O_4_@HPU-9** in different states: (1) **HPU-9**; (2) **HPU-9** absorption after one cycle; (3) **HPU-9** desorption after one cycle; (4) **R6G/HPU-9** absorption after one cycle; (5) **R6G/HPU-9** desorption after one cycle; (6) **R6G/HPU-9** absorption after four cycles.

Three cationic dyes, namely, MB, RhB and R6G, with different sizes and charges, and three anionic dyes, namely, MO, OG and NGB, with different sizes and charges were selected. The molecular structures of dyes are shown in Figure S2. The selected dyes displayed characteristic peaks. In particular, the main absorption peaks of MB, RhB, R6G, MO, OG and NGB were at 650, 550, 525, 466, 490 and 720 nm, respectively. In brief, 10 mg of freshly prepared **Fe_3_O_4_@HPU-9** crystals was immersed in the different aqueous solutions of the dyes (4 mg/L, 5 mL) at room temperature. All mixtures were placed in the dark and stirred for 24 h. UV-visible (UV-Vis) spectroscopy was performed to determine the absorption abilities of **Fe_3_O_4_@HPU-9**. As shown in Figure 5, the disappearance absorption peaks of the cationic dyes indicated that **Fe_3_O_4_@HPU-9** preferred to absorb the cationic dyes (MB, RhB and R6G) over a period of time. By contrast, the absorption of the anionic dyes (OG, MO and NGB) was not detected. So **Fe_3_O_4_@HPU-9** exhibited different absorption abilities toward different types of organic dyes (The self-changes of the cationic dye solution after 24 h were unchanged shown in Figure S3). Moreover, we studied the selective absorption and separation of cationic dyes from other mixtures, namely, MB & MO, MB & OG, MB & NGB, R6G & MO, R6G & OG, R6G & NGB, RhB & MO, RhB & OG, RhB & NGB. As shown in Figure 6 and Figure S4, after absorption for 24 h, the absorption peaks of the cationic dyes at their respective peaks were weakened, whereas those of the anionic dyes remain unchanged. It is observed that cationic dyes could be separated from other anionic dyes in aqueous solution by **Fe_3_O_4_@HPU-9** (Fe_3_O_4_ has no selectivity). In addition, the selective absorption and separation of the different cationic dyes (MB, R6G or RhB) from RhB & MB, R6G & MB, R6G & RhB were also studied (Figure S5). We found that **Fe_3_O_4_@HPU-9** did not exhibit a significant selectivity toward different cationic dyes. Therefore, we concluded that the distinguishing characteristics of the absorption behavior of **Fe_3_O_4_@HPU-9** toward dyes were possibly due to the structural traits of **Fe_3_O_4_@HPU-9** instead of size effect, specifically, the strong electrostatic affinity of the anionic channel of **Fe_3_O_4_@HPU-9** toward cationic dyes. PXRD confirmed that **Fe_3_O_4_@HPU-9** almost retained its framework structure during dye absorption (Figure 7), implying that the successful absorption of cationic dyes did not influence the integrity of the crystalline structure of **Fe_3_O_4_@HPU-9**. Therefore, **Fe_3_O_4_@HPU-9** can be developed as a potential adsorbent for removing cationic dyes in aquatic environments.

### 3.4. Absorption Kinetics and Absorption Isotherms

**Figure 8:**
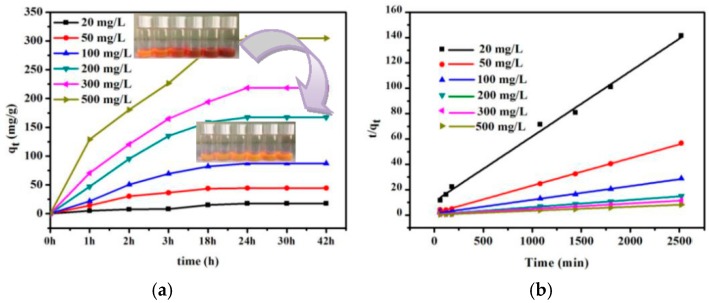
(**a**) The absorption isotherms for R6G absorption by **Fe_3_O_4_@HPU-9**, *C_e_*: equilibrium concentration of R6G, *q_e_*: the amount of R6G absorbed; (**b**) plots of pseudo-second-order kinetics for the absorption of R6G by **Fe_3_O_4_@HPU-9**.

The absorption experiment was run as follows: 10 mg of **Fe_3_O_4_@HPU-9** was immersed in 10 mL of R6G aqueous solutions. The data showed that the absorption capacities of the **Fe_3_O_4_@HPU-9** increased with the increase in the concentration of R6G (20, 50, 100, 200, 300, 500 M). As shown in Figure 8a, the absorption capacity for R6G significantly increased in the initial 18 h, gradually reaching equilibrium. Then, a pseudo-second-order model was explored for further exploration of the absorption kinetics. The constants were calculated by using the following equation [25]:

$$\frac{t}{q_{t}}=\frac{1}{q_{e}^{2}k_{2}}+\frac{t}{q_{e}}$$

where *q_t_* and *q_e_* are the amounts of R6G adsorbed at a given time and at equilibrium, respectively, and *k*_2_ is the rate constant for pseudo-second-order kinetics of R6G. As can be seen in Table 1 and Figure 8b, the fitting of the experimental results shows that the values obtained from the model fitting agree with the experimental data. The calculated values of *k*_2_ for the absorption of R6G are comparative to the previously reported MOF-235 (0.000218 g·mg^−1^·min^−1^). 

**Figure 9:**
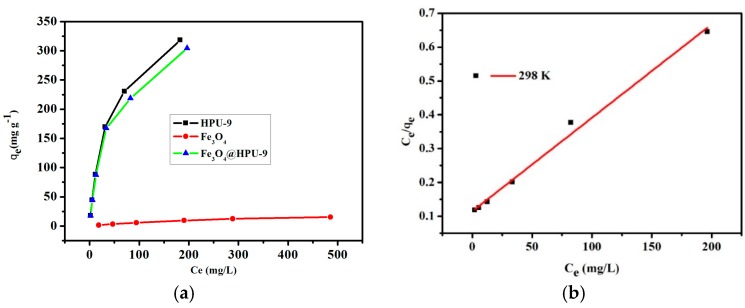
(**a**) Absorption isotherms for R6G by **HPU-9**, Fe_3_O_4_ and **Fe_3_O_4_@HPU-9**; (**b**) Langmuir plots of the isotherms for R6G absorption onto **Fe_3_O_4_@HPU-9**.

Absorption isotherms are also applied to describe the interaction between R6G and **Fe_3_O_4_@HPU-9**. Figure 9a shows the absorption isotherms of R6G onto Fe_3_O_4_ and **HPU-9** and **Fe_3_O_4_@HPU-9**. The absorption data were analyzed with the Langmuir equation [26]:

$$\frac{C_{e}}{q_{e}}=\frac{C_{e}}{Q_{m}}+\frac{1}{K_{L}Q_{m}}$$

where *C_e_*, *q_e_*, *Q_m_* and *K_L_* are the equilibrium concentration of R6G, the equilibrium absorption capacity, the maximum absorption capacity and the Langmuir constant, respectively. Table 2 shows the detailed data obtained with this analysis. The maximum absorption capacity of **Fe_3_O_4_@HPU-9** for R6G was 362.318 mg·g^−1^. *C_e_*/*q_e_* plotted against *C_e_* yielded straight lines, as shown in Figure 9b and Figure S6. The correlation coefficients, R^2^, of the Langmuir equation were found to be larger than 0.99, indicating the absorption of R6G follows the Langmuir absorption model.

**Figure 10:**
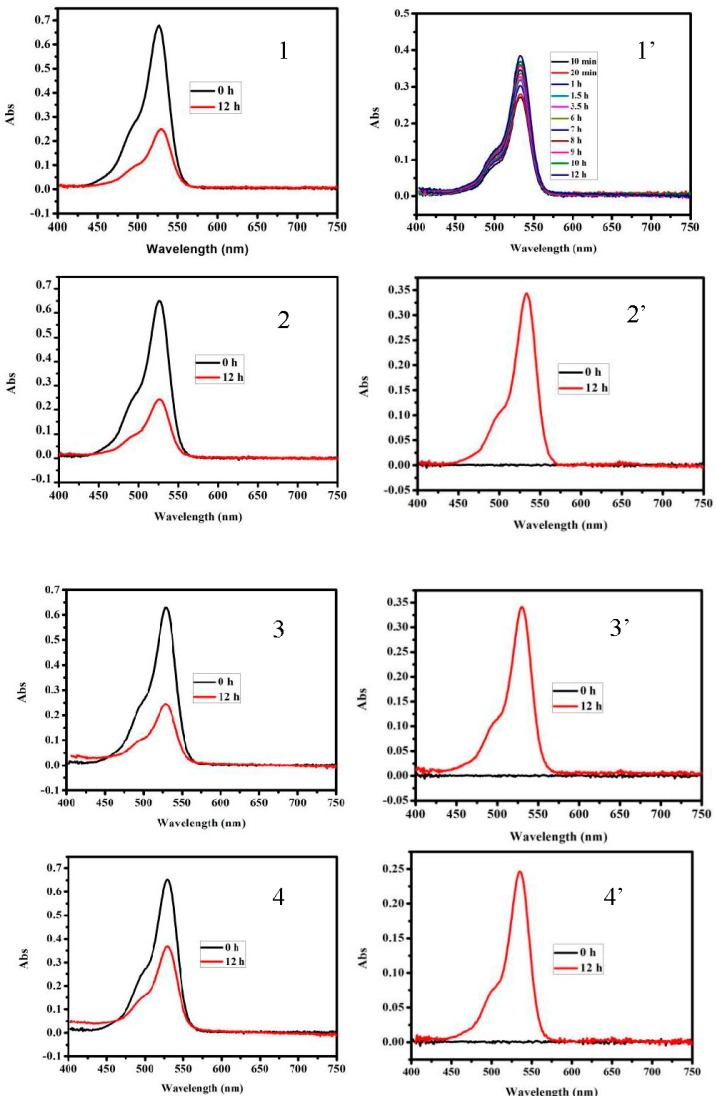
The four cycles of R6G absorption and desorption by **Fe_3_O_4_@HPU-9**.

A reusability test was performed to analyse the recyclable and reusable properties of **Fe_3_O_4_@HPU-9** (Figure 10). Under room temperature, the regeneration of **Fe_3_O_4_@HPU-9** was successfully achieved by immersing **R6G@Fe_3_O_4_@HPU-9** in saturated ethanol and NaCl for 12 h. The removal efficiency during the fourth cycle exhibited an almost similar rate as that of the first absorption cycle, indicating good regeneration and reusability. In the entire recycling process, the solution was poured directly without centrifugal separation. Our data confirmed that the proposed magnetic composite could be recycled and reused several times, making it more convenient, economic and efficient than common adsorbents.

## 4. Conclusions

In summary, we successfully synthesized a novel magnetic **Fe_3_O_4_@HPU-9** hybrid, which shows excellent absorption of cationic dyes from aqueous solution and could be easily separated and reused several times without degrading the absorption capacity. The **Fe_3_O_4_@HPU-9** particle possessed a well-defined core-shell structure consisting of a Fe_3_O_4_ core and a **HPU-9** shell. In the composites, the anionic **HPU-9** shell exhibits the selective absorption of cationic dyes by the utilization of the large pore space with anionic binding sites through direct electrostatic attractions between **HPU-9** and cationic dyes, and the Fe_3_O_4_ core serves as a magnetic particle. The maximum absorption capacity of **Fe_3_O_4_@HPU-9** for R6G was 362.318 mg·g^−1^. In the entire recycling process of dye absorption, the superabundant solution of dye was poured directly without centrifugal separation. Our data suggested that the magnetic **Fe_3_O_4_@HPU-9** hybrid has great potential to be employed as a convenient, economic and efficient adsorbent for the treatment of wastewater containing cationic dyes.

## Figures and Tables

**Table 1:** Kinetic parameters for the absorption of R6G by **Fe_3_O_4_@HPU-9** at 293K.

Pseudo-Second-Order	20 M	50 M	100 M	200 M	300 M	500 M
*q_e_* (mg·g^−1^)	19.48	46.18	92.42	175.74	229.35	316.45
R^2^	0.99504	0.99887	0.99724	0.99825	0.99745	0.99941
*K*_2_ (g·mg^−1^·min^−1^)	2.43 × 10^−4^	2.86 × 10^−4^	9.26 × 10^−5^	5.45 × 10^−5^	3.87 × 10^−5^	3.74 × 10^−5^

**Table 2:** Langmuir parameters for the absorption of R6G by **HPU-9**, Fe_3_O_4_ and **Fe_3_O_4_@HPU-9**.

Analyte	*q_exp_*	Langmuir Constants		
		*Q_m_*	*K_L_*	R^2^
**HPU-9**	317.273	384.615	0.025	0.99736
Fe_3_O_4_	15.377	25.013	0.0032	0.99159
**Fe_3_O_4_@HPU-9**	304.536	362.318	0.024	0.99151

## Supplementary Materials

The following are available online at http://www.mdpi.com/1996-1944/11/5/744/s1, Figure S1: The TG curve of **HPU-9 and Fe_3_O_4_@HPU-9**, Figure S2: The molecular structures of dyes, Figure S3: The self-changes of cationic dye solution after 24 h, Figure S4: The selective adsorption of cationic dye from the mixtures of cationic and anionic dye solutions by **Fe_3_O_4_@****HPU-9**, Figure S5: The adsorption competition between two kinds of cationic dyes by **Fe_3_O_4_@****HPU-9**., Figure S6: Langmuir plots of the isotherms for R6G adsorption onto Fe_3_O_4_ and **HPU-9**. Table S1: The information of regents, Table S2: Crystal data and structure refinement for **HPU-9** ^a^.

## References

[1] Yagub M.T., Sen T.K., Afroze S., Ang H.M. (2014). Polyethylenimine nanofibrous adsorbent for highly effective removal of anionic dyes from aqueous solution. Adv. Colloid Interface Sci..

[2] Jia Y.Y., Ren G.J., Li A.L., Zhang L.Z., Feng R., Zhang Y.H., Bu X.H. (2016). Temperature-Related Synthesis of Two Anionic Metal–Organic Frameworks with Distinct Performance in Organic Dye Absorption. Cryst. Growth Des..

[3] Robson K.C.D., Hu K., Meyer G.J., Berlinguette C.P. (2013). Atomic Level Resolution of Dye Regeneration in the Dye-Sensitized Solar Cell. J. Am. Chem. Soc..

[4] Yao Z.Y., Zhang M., Wu H., Yang L., Li R.Z., Wang P. (2015). Donor/Acceptor Indenoperylene Dye for Highly Efficient Organic Dye-Sensitized Solar Cells. J. Am. Chem. Soc..

[5] Gao M.L., Wang W.J., Liu L., Han Z.B., Wei N., Cao X.M., Yuan D.Q. (2017). Microporous Hexanuclear Ln(III) Cluster-Based Metal–OrganicFrameworks: Color Tunability for Barcode Application and Selective Removal of Methylene Blue. Inorg. Chem..

[6] Sharifzade G., Asghari A., Rajabi M. (2017). Highly effective adsorption of xanthene dyes (rhodamine B and erythrosine B) from aqueous solutions onto lemon citrus peel active carbon: Characterization, resolving analysis, optimization and mechanistic studies. RSC Adv..

[7] Takeshita J., Hasegawa Y., Yanai K., Yamamoto A., Ishii A., Hasegawa M., Yamanaka M. (2017). Organic Dye Absorption by Amphiphilic Tris-Urea Supramolecular Hydrogel. Chem. Asian J..

[8] Das P.P., Agarkar S.A., Mukhopadhyay S., Manju U., Ogale S.B., Devi P.S. (2014). Defects in Chemically Synthesized and Thermally Processed ZnO Nanorods: Implications for Active Layer Properties in Dye-Sensitized Solar Cells. Inorg. Chem..

[9] Sonu K.P., Pavan Kumar B.V.V.S., George Subi J., Eswaramoorthy M. (2017). Simple and Facile Approach to Create Charge Reversible Pores via Hydrophobic Anchoring of Ionic Amphiphiles. ACS Appl. Mater. Interfaces.

[10] Dong Z.Y., Sun Y.Z.S., Chu J., Zhang X.Z., Deng H.X. (2017). Multivariate Metal–Organic Frameworks for Dialing-in the Binding and Programming the Release of Drug Molecules. J. Am. Chem. Soc..

[11] Lv L.L., Yang J., Zhang H.M., Liu Y.Y., Ma J.F. (2015). Metal-Ion Exchange, Small-Molecule Sensing, Selective Dye Absorption, and Reversible Iodine Uptake of Three Coordination Polymers Constructed by a New Resorcin[4]arene-Based Tetracarboxylate. Inorg. Chem..

[12] Abdelhamid H.N., Huang Z.H., El-Zohry A.M., Zheng H.Q., Zou X.D. (2017). A Fast and Scalable Approach for Synthesis of Hierarchical Porous Zeolitic Imidazolate Frameworks and One-Pot Encapsulation of Target Molecules. Inorg. Chem..

[13] Lian X., Yan B. (2016). A lanthanide metal-organic framework (MOF-76) for adsorbing dyes and fluorescence detecting aromatic pollutants. RSC Adv..

[14] Zhao H.X., Zou Q., Sun S.K., Yu C.S., Zhang X.J., Li R.J., Fu Y.Y. (2016). Theranostic Metal-Organic Framework Core-Shell Composites for Magnetic Resonance Imaging and Drug Delivery. Chem. Sci..

[15] Dolatkhah A., Wilson L.D. (2016). Magnetite/Polymer Brush Nanocomposites with Switchable Uptake Behavior toward Methylene Blue. ACS Appl. Mater. Interfaces.

[16] An H.Y., Hu Y., Wang L., Zhou E.L., Fei F., Su Z.M. (2015). 3D Racemic Microporous Frameworks and 3D Chiral Supramolecular Architectures Based on Evans–Showell-Type Polyoxometalates Controlled by the Temperature. Cryst. Growth Des..

[17] Du P.Y., Gu W., Liu X. (2016). Multifunctional Three-Dimensional Europium Metal–Organic Framework for Luminescence Sensing of Benzaldehyde and Cu^2+^ and Selective Capture of Dye Molecules. Inorg. Chem..

[18] Dey A., Konavarapu S.K., Sasmal H.S., Biradha K. (2016). Porous Coordination Polymers Containing Pyridine-3,5-Bis(5-azabenzimidazole): Exploration of Water Sorption, Selective Dye Absorption, and Luminescent Properties. Cryst. Growth Des..

[19] Song Y., Fan R.Q., Xing K., Du X., Su T., Wang P., Yang Y.L. (2017). Insight into the Controllable Synthesis of Cu(I)/Cu(II) Metal–Organic Complexes: Size-Exclusive Selective Dye Absorption and Semiconductor Properties. Cryst. Growth Des..

[20] Lu L., Wu J., Wang J., Liu J.Q., Li B.H., Singh A., Kumard A., Batten S.R. (2017). An uncommon 3D 3, 3, 4, 8-c Cd(II) metal-organic framework for highly efficient luminescent sensing and organic dye absorption: Experimental and theoretical insight. CrystEngComm.

[21] Ke F., Yuan Y.P., Qiu L.G., Shen Y.H., Xie A.J., Zhu J.F., Tian X.Y., Zhang L.D. (2011). Facile fabrication of magnetic metal–organic framework nanocomposites for potential targeted drug delivery. J. Mater. Chem..

[22] Xiong Y.H., Ye F.G., Zhang C., Shen S.F., Su L.J., Zhao S.L. (2015). Synthesis of magnetic porous γ-Fe_2_O_3_/C@HKUST-1 composites for efficient removal of dyes and heavy metal ions from aqueous solution. RSC Adv..

[23] Li L., Liu X.L., Gao M., Hong W., Liu G.Z., Fan L., Hu B., Xia Q.H., Liu L., Song G.W. (2014). The absorption on magnetic hybrid Fe_3_O_4_/HKUST-1/GO of methylene blue from water solution. J. Mater. Chem. A.

[24] Zhao M., Zhang X.M., Deng C.H. (2015). Rational synthesis of novel recyclable Fe_3_O_4_@MOF nanocomposites for enzymatic digestion. Chem. Commun..

[25] Huo S., Yan X. (2012). Metal–organic framework MIL-100(Fe) for the absorption of malachite green from aqueous solution. J. Mater. Chem..

[26] Zhao X., Liu D., Huang H., Zhang W., Yang Q., Zhong C. (2014). The stability and defluoridation performance of MOFs in fluoride solutions. Microporous Mesoporous Mater..

