# Supplementary material for: Facile Fabrication of Magnetic Metal-Organic Framework Composites for the Highly Selective Removal of Cationic Dyes
Source: Materials (Basel). 2018 May 7;11(5):744. doi: 10.3390/ma11050744 (PMC5978121; doi:10.3390/ma11050744)
Supplement: Supplementary file 1 [file materials-11-00744-s001.pdf]

Facile fabrication of magnetic metal-organic framework composites for the highly selective removal of cationic dyes

Huijun Li, Qingqing Li,Yaling He, Ning Zhang, Zhouqing Xu, Yuan Wang**

College of Chemistry and Chemical Engineering, Henan Polytechnic University, Jiaozuo, Henan 454000, China

Corresponding author: zhqxu@hpu.edu.cn;wangyuan08@hpu.edu.cn;

Table S1 The information of regents.

reagents	purity	company
$Cd(NO_3)_2·4H_2O$	99%	aladdin
$FeCl_3·6H_2O$	99%	aladdin
CH_3OH	99.8%	aladdin
CH_3CN	99%	aladdin
NaAc	99.99%	aladdin
DMA	99%	aladdin
MO (Methyl Orange)	95%	*J&K*
MB (Methylene blue)	97%	aladdin
R6G (Rhodamine 6G)	95%	aladdin
OG (Orange G)	98%	aladdin
NGB (Naphthol Green B)	98%	aladdin
RhB (Rhodamine B)	98%	aladdin

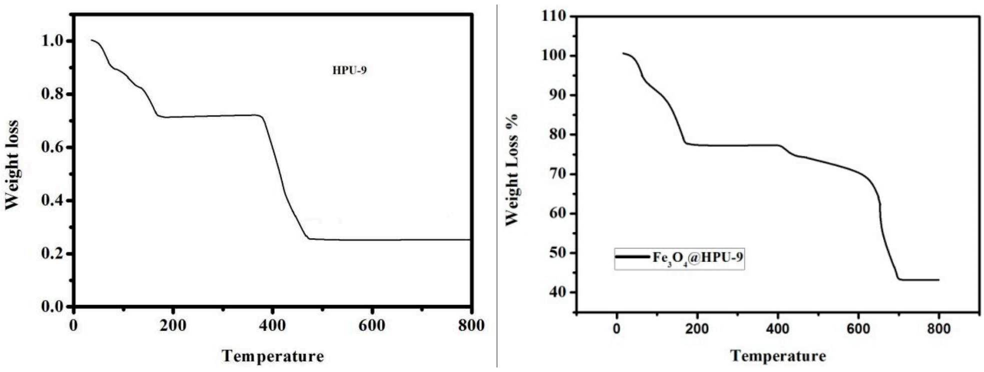

Figure S1 The TG curve of **HPU-9 and Fe_3O_4@HPU-9**.

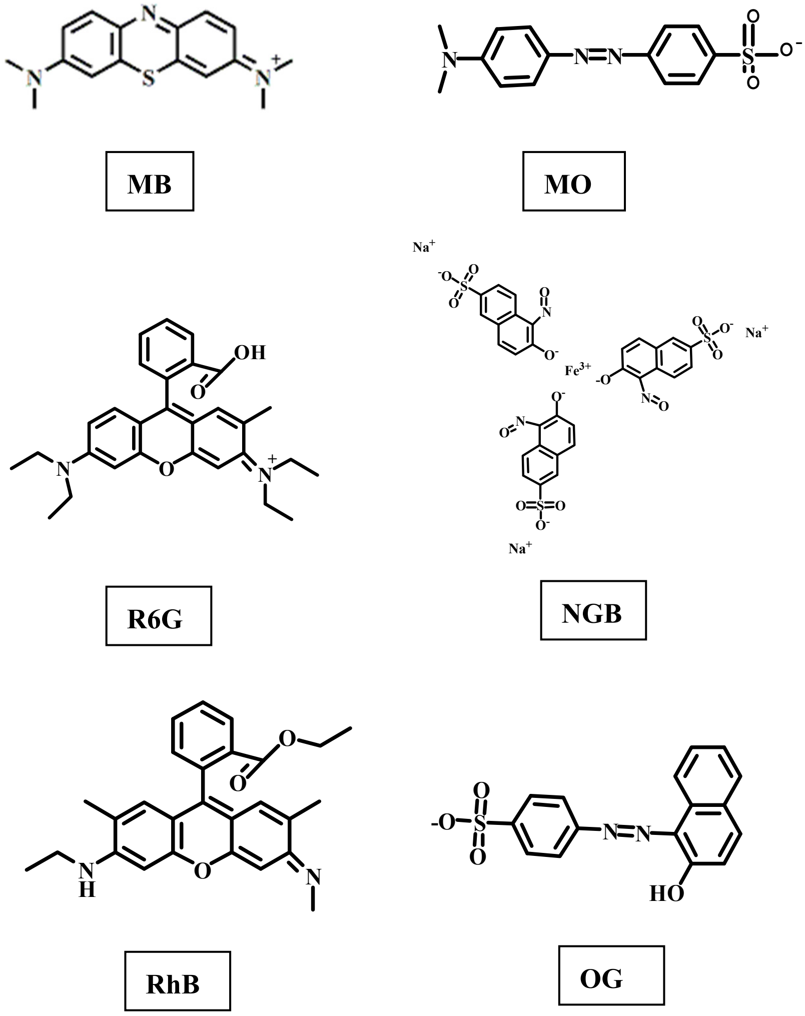

Figure S2 The molecular structures of dyes.

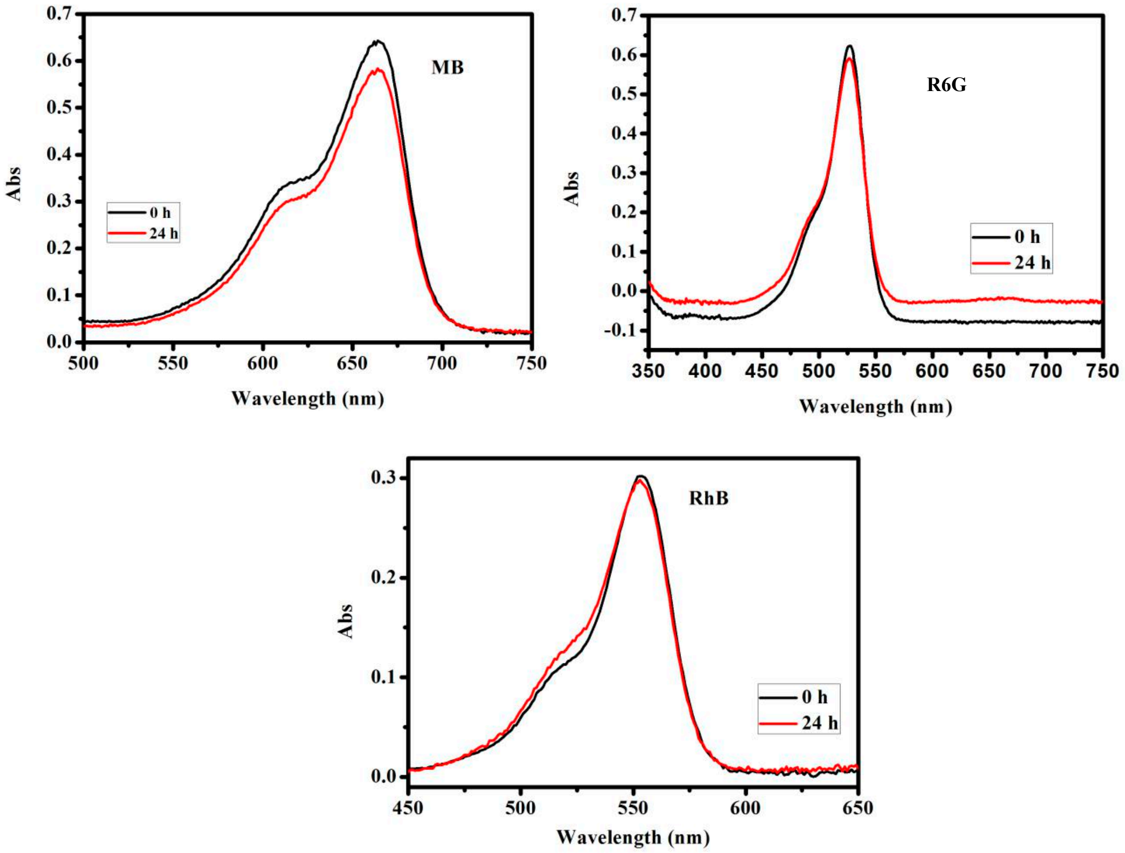

Figure S3 The self-changes of cationic dye solution after 24 h.

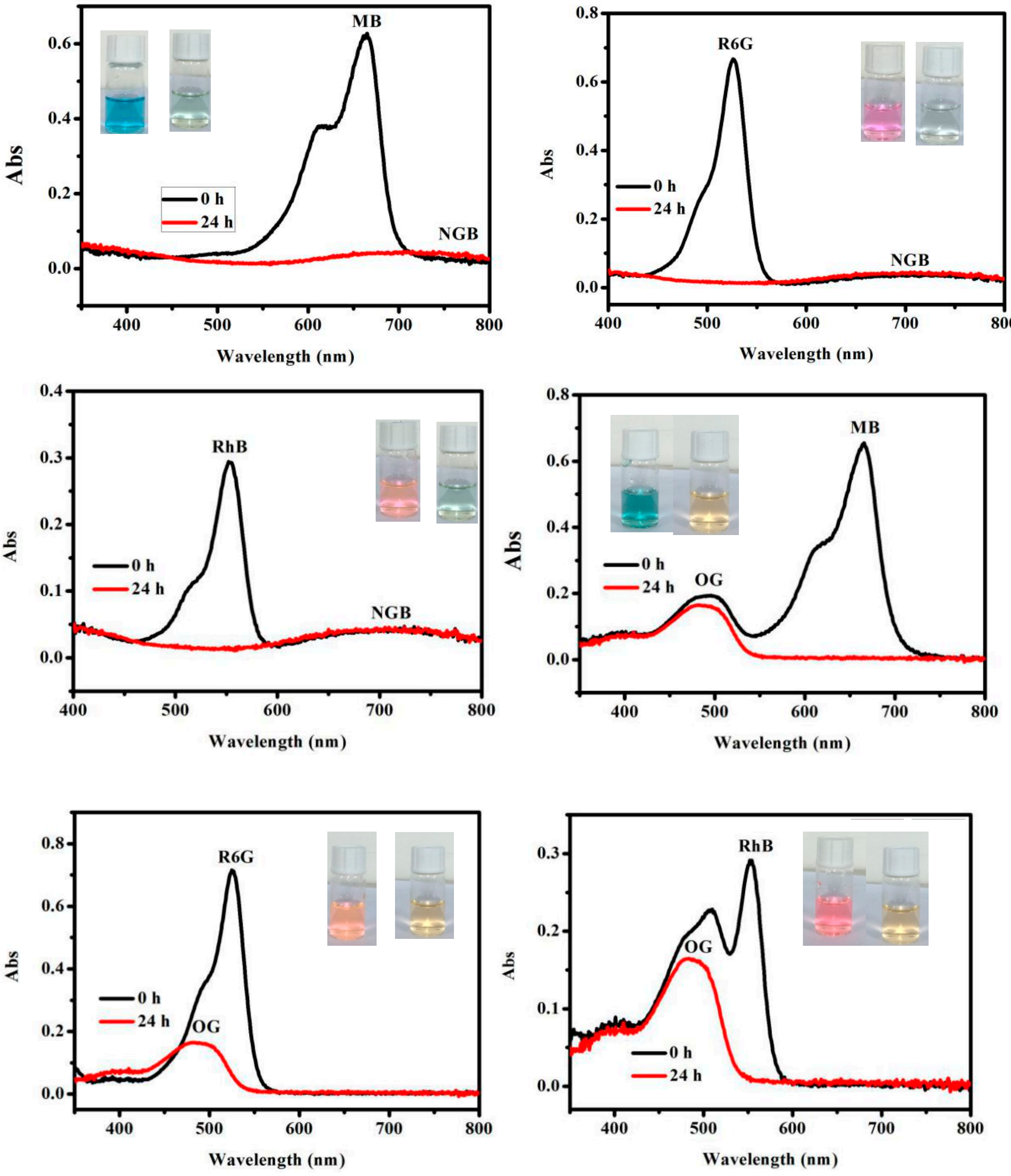

Figure S4 The selective adsorption of cationic dye from the mixtures of cationic and anionic dye solutions by **Fe_3O_4@HPU-9**.

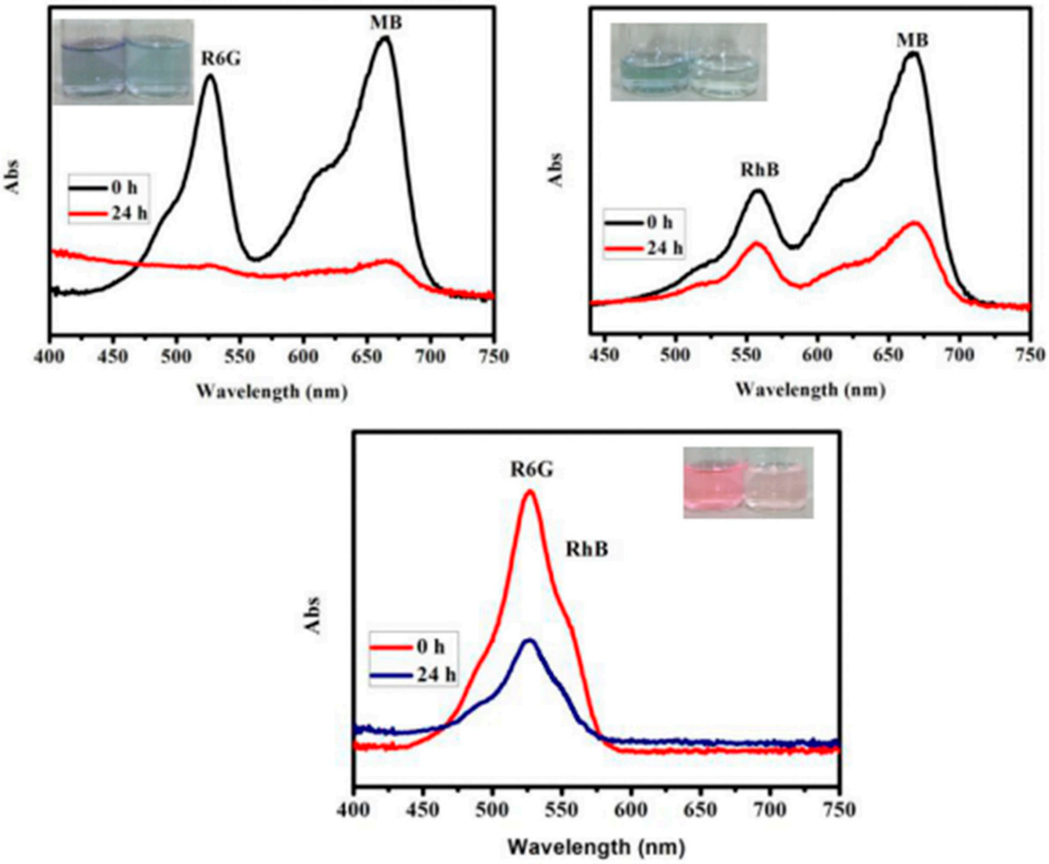

Figure S5 The adsorption competition between two kinds of cationic dyes by **Fe_3O_4@HPU-9**.

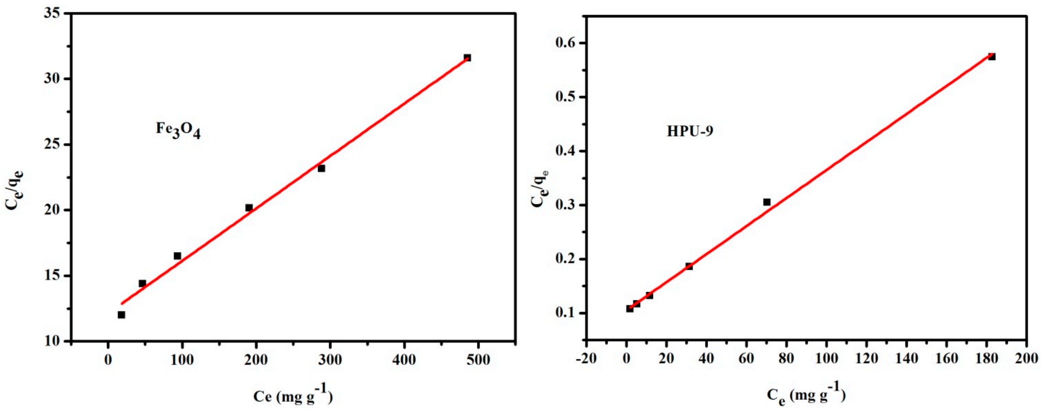

Figure S6 Langmuir plots of the isotherms for R6G adsorption onto Fe_3O_4 and **HPU-9**.

Table S2. Crystal data and structure refinement for **HPU-9**[a]

	**HPU-9**
Formula	$C_{18}H_{21}CdN_4O_6$
Fw	501.79
temp/K	296(2)
Wavelength(Å)	0.71073
crystal system	orthorhombic
space group	*P c c n*
a (Å)	11.814(4)
b (Å)	16.518(6)
c (Å)	18.452(7)
α/β/γ/deg	90
V (Å^3)	3601(2)
Z	8
D_c(mg·m^{-3})	1.851
F (000)	2024
rflns collected	4777
unique rflns	1947
GOF on F^2	1.051
R_1[a](I>2sigmaI	0.0559
wR$_2$[b] (I>2sigmal)	0.1235
R_1(all data)	0.1557
wR$_2$	0.1437

[a] $R_1 = ||F_o| - |F_c|| / |F_o|$. [b] $wR_2 = [w(F_o^2 - F_c^2)^2/w(F_o^2)^2]^{1/2}$.
